# Chemical Composition, Antioxidant and α-Glucosidase-Inhibiting Activities of the Aqueous and Hydroethanolic Extracts of *Vaccinium myrtillus* Leaves

**DOI:** 10.3390/molecules22050703

**Published:** 2017-04-28

**Authors:** Kristina Bljajić, Roberta Petlevski, Lovorka Vujić, Ana Čačić, Nina Šoštarić, Jasna Jablan, Isabel Saraiva de Carvalho, Marijana Zovko Končić

**Affiliations:** 1 Faculty of Pharmacy and Biochemistry, University of Zagreb, A. Kovačića 1, 10000 Zagreb, Croatia; gasparkristina@yahoo.com (K.B.); rpetlevski@pharma.hr (R.P.); lvujic@pharma.hr (L.V.); anacacicka@hotmail.com (A.Č.); nina.sostaric@gmail.com (N.Š.); jjablan@pharma.hr (J.J.); 2 Faculty of Sciences and Technology, University of Algarve, Campus de Gambelas, Bd. 8, 8005-139 Faro, Portugal; icarva@ualg.pt

**Keywords:** α-glucosidase inhibition, antioxidant activity, polyphenol, glutathione, *Vaccinium myrtillus*

## Abstract

*Vaccinium myrtillus* (bilberry) leaf is traditionally used in southeastern Europe for the treatment of diabetes. In the present study, the ability of bilberry leaf extracts to inhibit carbohydrate-hydrolyzing enzymes and restore glutathione concentration in Hep G2 cells subjected to glucose-induced oxidative stress was investigated. A comprehensive analysis of the antioxidant activity of two bilberry leaf extracts was performed. The aqueous extract showed excellent total antioxidant and chelating activity. Its antioxidant activity in the β-carotene-linoleic acid assay was very good, reaching the activity of the antioxidant standard BHA (93.4 ± 2.3% vs. 95.1 ± 2.4%, respectively). The hydroethanolic extract (ethanol/H_2_O, 8:2, *v*/*v*), on the other hand, was a better radical scavenger and Fe^2+^ reducing agent. Furthermore, the aqueous extract was able to efficiently increase glutathione concentration in Hep G2 cells subjected to glucose-induced oxidative stress and restore it to the levels observed in non-hyperglycaemic cells. The hydroethanolic extract strongly inhibited α-glucosidase, with the IC_50_ statistically equal to the antidiabetic drug acarbose (0.29 ± 0.02 mg/mL vs. 0.50 ± 0.01 mg/mL, respectively). Phytochemical analysis revealed the presence of quercetin and kaemferol derivatives, as well as chlorogenic and *p*-coumaric acid. The study results indicate that *V. myrtillus* leaf may have promising properties as a supporting therapy for diabetes.

## 1. Introduction

Reactive oxygen species (ROS) have many important functions in normal metabolism, however, excess ROS can produce oxidative damage to cellular macromolecules, impair protein function, and trigger cell death. Some medical disorders lead to overproduction of ROS and a constant state of enhanced oxidative stress. Among them, the most prominent are metabolic syndrome and type 2 diabetes [1] which affect over 30–40% of the population older than 65 [2]. Those ailments are characterized by insulin resistance and, consequently, hyperglycemia. Hyperglycemia causes increased flux of glucose through the polyol and hexosamine pathways, increased formation of advanced glycation end-products, activation of protein kinase C, mitochondrial dysfunction, and consequently ROS accumulation. This leads to cellular damage and the development of diabetic complications such as neuropathy, nephropathy and retinopathy, as well as to liver damage [1,3].

In order to diminish the consequences of hyperglycemia-induced oxidative stress, human organism utilizes various endo- and exogenous antioxidants. Glutathione (GSH) is one of the most important cellular non-protein antioxidants. This tripeptide is a co-factor in several important enzymatic reactions, including those involved in detoxification of xenobiotics [4,5]. In states of increased ROS production, such as diabetes, levels of glutathione in cells may deplete thus further aggravating ROS accumulation. Some natural antioxidants, such as curcumin or sulforaphane [4] may change the GSH status and prevent consequences of diabetes and metabolic syndrome [6]. Besides their influences on GSH content, natural substances can exert other biological activities, beneficial in states of elevated oxidative stress. For example, in vitro and in vivo studies indicate that, due to their direct antioxidant activity or via their influence on endogenous antioxidants, natural polyphenols could protect cellular targets in the eye, kidney or liver that are susceptible to diabetic complications [1,7].

In addition to their antioxidant activity, natural products and extracts can influence the enzymes participating in carbohydrate metabolism. For example, they can impair starch digestion by inhibiting α-amylase, an enzyme secreted in saliva and pancreatic juice which catalyzes the hydrolysis of starch to a mixture of smaller oligosaccharides. Another enzyme whose inhibition can retard the postprandial increase of glucose concentration is α-glucosidase, enzyme responsible for digestion of oligosaccharides to glucose. It has been repeatedly shown that medicinal plants are a rich source of α-amylase and α-glucosidase inhibitors and there is hope that they can be helpful in the treatment and prevention of diabetes, obesity and hyperlipemia [8].

As the interest in the therapeutic benefits of herbal products increases, the concern over the safety and toxicity of natural herbs and formulations available the market is also growing [9]. In addition to phytochemicals with beneficial health-related effects, herbs may also contain harmful substances, such as heavy metals, pathologic microorganisms or pesticides. Before coming to the market, herbal supplements and similar products are analyzed for their health safety, which usually comprises the analysis of heavy metal content. However, herbal material bought at local herbalists is generally not subjected to the rigorous quality control by authorities and often contains significant amount of toxic metals [10,11], which may adversely affect the consumers’ health [9].

The use of *Vaccinium myrtillus* L., (bilberry, Ericaceae) leaf for diabetes was widespread at the beginning of 20th century before the discovery of insulin as one of the most frequently used herbal antidiabetic remedies. Even though clinical trials failed to unequivocally demonstrate its hypoglycaemic activity [12], many different bilberry leaf extracts, sold either as dry extracts or hydroalcoholic tinctures, are available on the market. In addition to that, recent surveys of traditionally used antidiabetics in Croatia [13] and Bosnia and Herzegovina [14], revealed that bilberry leaf tea is still recommended and sold by more than 40% of the herbalists for the management of diabetes. Having in mind that, besides glucose-lowering effects, herbal extracts may have various other activities beneficial in the treatment of diabetes, the aim of this work was to evaluate phenolic composition, inhibitory effects against enzymes participating in carbohydrate digestion, antioxidant and GSH-protecting activities of aqueous and hydroethanolic (ethanol/H_2_O, 8:2, *v*/*v*) *V. myrtillus* L. extracts, as well as their safety regarding heavy metal content.

## 2. Results and Discussion

### 2.1. Phytochemical Analysis

Bilberry leaf is one of the most popular herbal remedies for diabetes, recommended and sold by local herbalists, often without appropriate health safety and quality control. In order to investigate the safety of the plant material used in this study, total reflection X-ray fluorescence (TXRF) was applied for the simultaneous multielemental analysis of plant extract. Ca (2940 ± 4.5 mg/kg) and K (2267 ± 4.9 mg/kg) were the dominant elements in the sample. Mn (474 ± 0.95 mg/kg), P (193.5 ± 7 mg/kg), Ba (51.1 ± 0.65 mg/kg), Zn (18.05 ± 0.1 mg/kg) existed in lower, but substantial quantities. Due to their toxicity, quantity of heavy metals in the herbal sample was of special interest. As (1.05 ± 0.05 mg/kg), Cu (7.1 ± 0.1 mg/kg), Fe (14.85 ± 1.5 mg/kg), Ni (0.2 ± 0.05 mg/kg) and Pb (0.55 ± 0.01 mg/kg) were detected but their concentrations were much lower than the legal threshold limit values established for the dietary supplements and much lower than in a previous study performed in Poland [15]. Even though there are reports that *V. myrtillus* leaf contains significant amounts of chromium, an essential element involved in the action of insulin [16], it was not detected in the analyzed sample. This result is therefore concurring with a study performed in Northern Europe [17]. Some authors consider chrome to be the main factor responsible for potential antidiabetic effects of *V. myrtillus* leaf [16]. However, its absence in the investigated material indicates that such opinion cannot be generalized to all *V. myrtillus* specimens.

**Figure 1:**
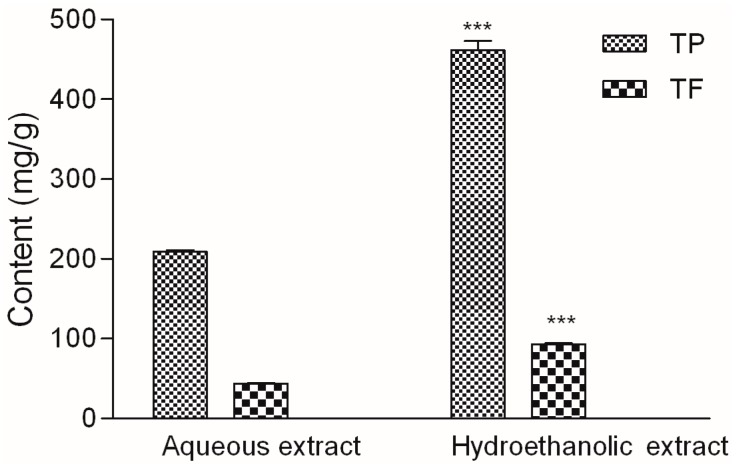
Total phenol (TP) and total flavonoid (TF) content in *V. myrtillus* extracts. Values are average of 3 replications ± SD. Statistical differences were assessed using *t*-test (*** *p* < 0.001).

Besides being sold in form of dry herbal material, bilberry leaf is often extracted, usually with water/ethanol mixtures, and sold either in form of a tincture or dry extract. However, water, ethanol and their combinations of varying ratios, have different polarity and viscosity interacting thus with different natural compounds, which is inevitably reflected in the biological properties of prepared extracts [18]. For the preparation of *V. myrtillus* leaf extracts in this study water and 80% (*v*/*v*) ethanol/water mixture were selected. The content of phenolic compounds in the prepared extracts is presented in Figure 1. The amount of total phenols and flavonoids in hydroethanolic extract was more than twofold higher than their amount in aqueous extract. This is expected because of the moderate polarity and good solubility of natural phenols in 80% ethanol.

**Figure 2:**
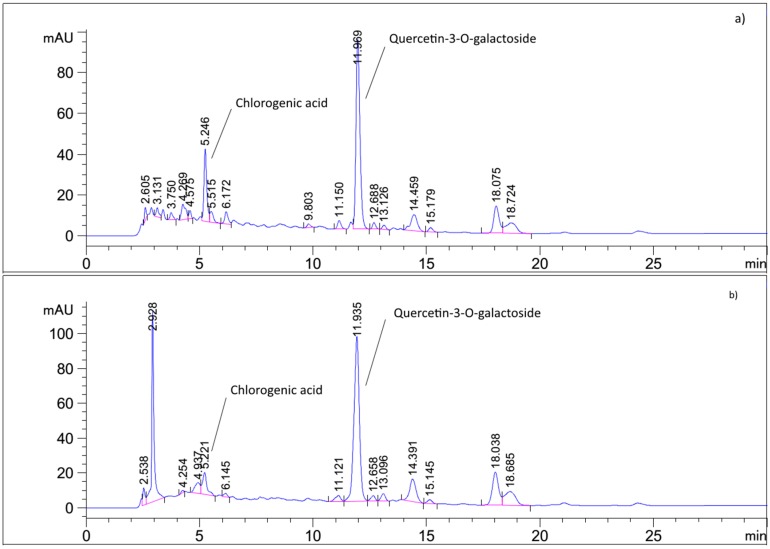
Chromatogram of aqueous (**a**) and hydroethanolic; (**b**) *V. myrtillus* extract recorded at 270 nm.

Results of HPLC analysis presented in Figure 2 and Table 1 revealed that the two extracts contained similar phenolic constituents but in different quantities. Quercetin-3-*O*-galactoside (hyperoside) was the dominant component in both extracts, followed by chlorogenic acid. In accordance with the solvent polarity, hydroethanolic extract contained somewhat more hyperoside, while the aqueous extract was richer in chlorogenic acid. Of the other two phenolic acids, *p*-coumaric acid was present in much lower amounts. A small amount of caffeic acid was detected only in the aqueous extract. In addition to that, kaempferol was detected in the hydrolyzed hydroethanolic extract, indicating the presence of its glycosides in the original extract, as reported before [16]. The presence of hyperoside, chlorogenic and *p*-coumaric acid in *V. myrtillus* leaf has been confirmed on many occasions [14,19,20]. Interestingly, arbutin was detected in the aqueous extract. Even though older literature allows for the possibility of arbutin presence, [16], now it is generally considered that arbutin content is caused by admixture with *Vaccinium* × *intermedium*, hybrid of *Vaccinium vitis-idaea* and *V*. *myrtillus*. However, a detailed morphological identification according to the specialized reference [21], excluded that possibility. Even though the amount of arbutin was relatively low, its presence in the investigated sample indicates that the excessive use of *V. myrtillus* leaf aqueous extracts (e.g., infusions) should be avoided.

As expected, quercetin was the main component of the hydrolyzed extracts. Its quantity, in accordance with the previously published studies, suggests that besides hyperoside there are other quercetin derivatives in the samples. High content of protocatechuic acid, a well-known product of flavonol degradation [22], in the aqueous extract, suggests that the degradation of quercetin occurred during the hydrolysis of aqueous extract.

### 2.2. Antioxidant Activity of the Extracts

Natural antioxidants may display their activity by numerous mechanisms. Antioxidants may influence oxidation reactions directly, through hydrogen atom transfer and single electron transfer, or indirectly, by their ability to bind potentially pro-oxidative metal ions [23]. The proportion of each of those mechanisms in total antioxidant activity of an herbal extract depends on various influences. Therefore, use of more than one method is recommended to give a comprehensive analysis of antioxidant efficiency of complex mixtures such as natural extracts. Due to the growing popularity of phenolic antioxidants, many scientific methods have been developed to measure antioxidant capacity of phenolics present in medicinal plants and foods. In order to systematically evaluate the antioxidant activity of *V. myrtillus* extracts in this study, multiple assay systems were used: total antioxidant activity, reducing power, ferric reducing antioxidant power, ABTS and DPPH radical scavenging and chelating activity assay. BHA, ascorbic acid, Trolox and EDTA, antioxidants and ion chelator often employed in the food and pharmaceutical industry, were used as the positive controls [24].

**Figure 3:**
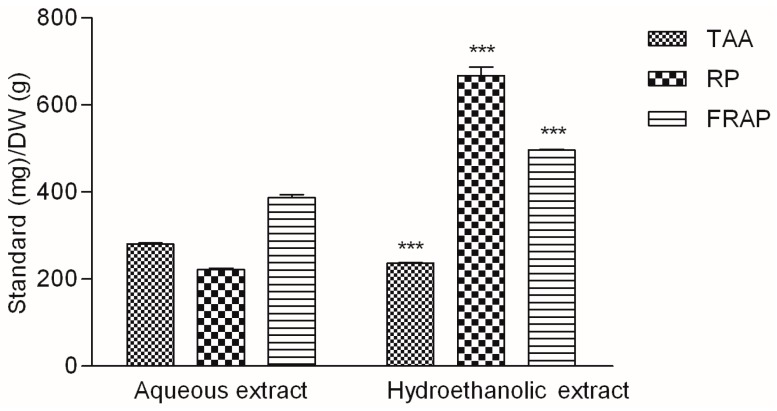
Total antioxidant activity (TAA), reducing power (RP) and ferric reducing antioxidant power assay (FRAP). Ascorbic acid (TAA) and Trolox (RP, FRAP) were used as standard antioxidants. Values are average of 3 replications ± SD. Statistical differences were assessed using *t-*test (*** *p* < 0.001).

Figure 3 presents the results of three assays based on the reducing abilities of the analytes. While the TAA assay is based on the reduction of Mo^6+^ to Mo^5+^ by the sample analyte [25], both RP and FRAP assays measure the ability of the extracts to reduce Fe^3+^ to Fe^2+^ ions. Even though the FRAP assay is based on the same process as RP assay, it is more specific than the previous one. The reducing properties of the extracts were not equally pronounced toward the two ions. The aqueous extract was more active in the TAA assay, while the reducing properties of the hydroethanolic extract were more pronounced in the reaction with iron ions, especially in the reducing power assay.

**Figure 4:**
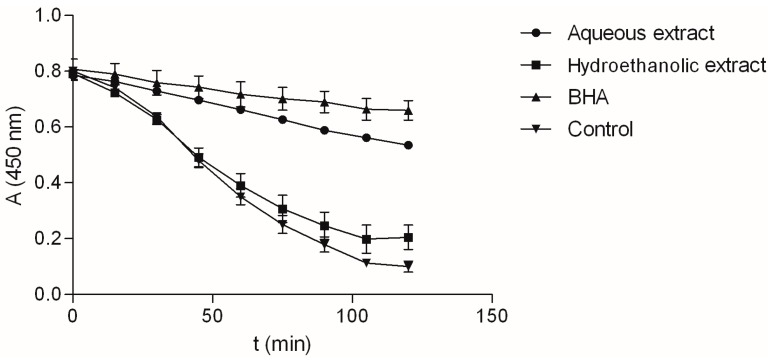
Decrease of absorbance at 450 nm in β-carotene-linoleic acid solution with the extracts or BHA. Values are average of 3 replications ± SD.

As shown in Table 2, the antioxidant activity of the investigated extracts was notable, but it differed between extracts. For example, the aqueous extract was very good inhibitor of β-carotene degradation (Figure 4), and its ANT activity was equal to the activity of BHA. Hydroethanolic extract on the other hand, displayed significant but much lower activity. Comparison of IC_50_ values shows that radical scavenging ability for ABTS free radical did not differ statistically between the two extracts. Although the hydroethanolic extract showed notably better DPPH radical scavenging capabilities than the aqueous extract, its activity was still somewhat lower than the activity of the commercial antioxidant, BHA. Finally, as evident by markedly lower IC_50_ values, aqueous extracts was shown to be better Fe^3+^ ion chelator than hydroethanolic extract and acts thus as a better secondary antioxidant.

In the presented study both investigated *V. myrtillus* leaf extracts were active in all the employed assays. They showed reducing activity (as evidenced by TAA, RP and FRAP assay), antiradical activity (in ABTS and DPPH free radical scavenging activity as well as in linoleic acid assay), as well as the potential to bind potentially pro-oxidant Fe^2+^ ion. As such, they could interact with ROS and other reactive species in multiple ways reducing thus their reactivity and deleterious properties. Such notable antioxidant activity of the extracts in the performed assays can mostly be related to their high phenolic content. However, similar to other studies investigating the relationship between antioxidant activity and phenolic content, a direct relationship could not be established. Various constituents interact in specific ways making each extract most suitable for different aspects of antioxidant activity [26].

### 2.3. Enzyme-Inhibitory Activity of the Extracts

**Figure 5:**
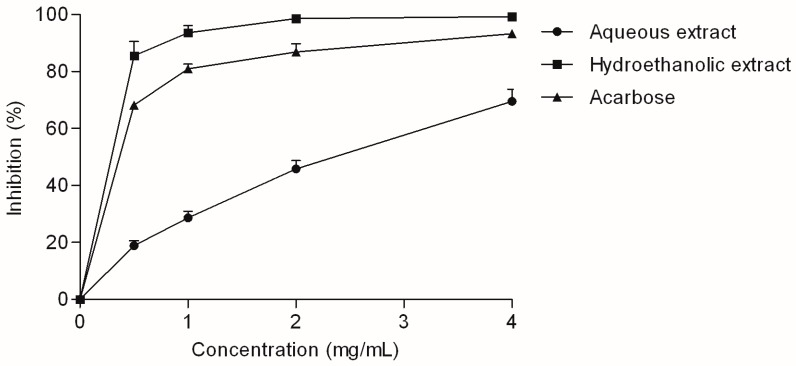
α-Glucosidase inhibitory activity of *V. myrtillus* extracts. Values are average of three replicates ± SD.

The investigated *V. myrtillus* extracts were tested for their inhibitory activity against two enzymes that participate in carbohydrate digestion: α-amylase and α-glucosidase. Although *V. myrtillus* leaf reportedly showed a positive response in previous screening of α-amylase inhibiting properties [16], the extracts in this study did not demonstrate any observable α-amylase-inhibiting effect. However, the α-glucosidase-inhibiting activity of the extracts was excellent (Figure 5).

The hydroethanolic extract was a particularly good α-glucosidase inhibitor, with an IC_50_ value statistically equal to the IC_50_ of acarbose, an anti-diabetic drug used as standard inhibitor (Table 2). It was previously reported that arbutin, hyperoside and chlorogenic acid, the main phenolic components of the investigated extracts, may suppress postprandial hyperglycemia by strongly inhibiting α-glucosidase [27,28,29]. Additionally, current studies show that the combination of plant phenolics, such as the combination found in the investigated extracts, may have an additive effect on α-glucosidase inhibition [30]. Furthermore, quercetin and caffeic acid, aglycones formed by hydrolysis of hyperoside and chlorogenic acid, respectively, also have notable anti-α-glucosidase activity [27,31]. Therefore, the observed α-glucosidase inhibitory activity may significantly contribute to the beneficial effects of V. myrtillus in diabetes.

### 2.4. Effect of V. myrtillus Extracts on Glucose-Induced Oxidative Stress in Hep G2 Cells

It was shown that the acute exposure to high glucose concentration produces oxidative stress in liver cells as evidenced by altered morphology, increased ROS level, lipid peroxidation, protein carbonyl and 3-nitrotyrosine adduct formation in Hep G2 cells. These changes may irreversibly damage hepatocytes leading to apoptosis. Natural metabolites and extracts may prevent the oxidative changes, normalize the concentration of intracellular antioxidants and thus prevent or even reverse cell-damage in vivo and in vitro [32]. Besides directly reacting with ROS, natural phenols and other antioxidants may interact with molecular targets in human organism by numerous other mechanisms [33]. One of the mechanisms by which antioxidants can protect cells and tissues from oxidative stress is regeneration of low molecular weight antioxidants, such as GSH [33]. The influence of *V. myrtillus* leaf extract on GSH levels in hepatic cells subjected to high glucose concentration has not been thoroughly investigated. Therefore, human hepatocellular carcinoma, Hep G2, cells were chosen as a model to study the potential effects of the prepared extracts on glucose-induced oxidative stress in liver.

**Figure 6:**
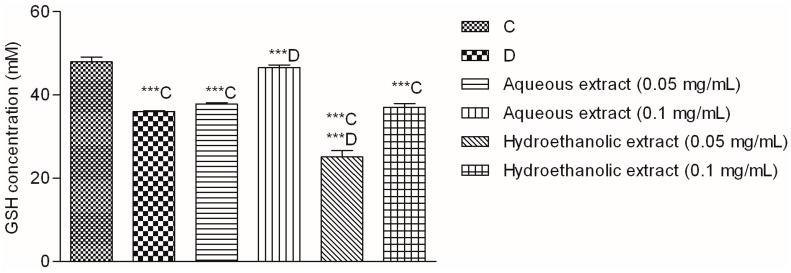
Glutathione (GSH) concentration in Hep G2 cells. C—Cells in MEM containing 5.56 mM glucose; D—Cells in MEM supplemented with additional 30 mM glucose. ***C and ***D—value statistically different from C and D, respectively (*p* < 0.001, Dunnett’s test). Values are average of 3 replications ± SD.

One of the consequences of diabetic hyperglycemia is an elevated production of ROS which leads to decreased level of one of the most important antioxidant in the body, GSH [34]. Indeed, in the performed assay the level of glutathione in glucose-treated (D) cells was significantly lower than in the non-treated cells (C) (Figure 6).

At the higher investigated concentration, the aqueous extract showed an outstanding activity and restored the GSH concentration to the levels observed in non-stressed cells (C). Quercetin derivatives and chlorogenic acid, the main components of the extract, have well documented in vitro antioxidant properties [35]. Furthermore, their antidiabetic activity has been confirmed in numerous in vivo studies. For example, treatment with hyperoside may prevent glomerular podocyte apoptosis in streptozotocin-induced diabetic nephropathy [36]. Moreover, hyperoside decreased albuminuria at the early stage of diabetic nephropathy by ameliorating renal damage and podocyte injury [37], improved the function of pancreatic islets, increased glycolysis and decreased gluconeogenesis in streptozotocin-induced diabetic rats [38]. Besides hyperoside, the extracts contained significant amounts of chlorogenic acid. This phenolic acid has well known antidiabetic properties which have been extensively reviewed [39] and linked to the observed diabetes protection of regular coffee consumption [40]. Furthermore, chlorogenic acid may ameliorate oxidative stress for renal injury in streptozotocin-induced diabetic nephropathy rats [41]. Thus we may conclude that phytochemical composition of *V. myrtillus* extracts is well suited for modulation of oxidative stress in hepatic cells. Even though the observed antioxidant and hepatoprotective effects of the extracts in Hep G2 model cannot be taken as a final proof that they would display such activity in the clinical setting, they certainly indicate a positive potential which will hopefully be researched further.

## 3. Materials and Methods

### 3.1. Plant Materials and Chemicals

Herbal material was bought from a herbalist at the market in Tuzla (Bosnia and Herzegovina) (44°32′21.9″ N 18°40′30.1″ E) in July 2012. It consisted of dry *V. myrtillus* leaves with stem parts. Plant material was identified by Professor Antun Alegro and Vedran Šegota, an expert associate of Herbarium Croaticum at Division of Botany, Department of Biology, Faculty of Science, University of Zagreb. A voucher specimen is deposited in the Department of Pharmacognosy, Faculty of Pharmacy and Biochemistry, University of Zagreb. For extraction, an ultrasonic bath (Bandelin SONOREX^®^ Digital 10 P DK 156 BP, Berlin, Germany) was used. Total Reflection X-ray Fluorescence (TXRF) measurements of plant extract samples were performed using a S2 Picofox benchtop TXRF spectrometer (Bruker Nano GmbH, Berlin, Germany). The instrument was equipped with a Mo target micro focus tube, operated at 50 kV/750 μA, a multilayer monochromator with 80% reflectivity and the liquid nitrogen-free XFlash^®^ silicon drift detector with an energy resolution of <150 eV (Mn Kα). Spectroscopic measurements were performed using a T70+ UV/Vis spectrometer (PG Instruments Ltd., Leicestershire, UK) and a Stat Fax 3200 microplate reader (Awareness Technologies, Westport, CT, USA). Contents of individual flavonoids and phenolic acids were determined using an HPLC instrument (Agilent 1200 series, Agilent Technologies, Santa Clara, CA, USA) equipped with an autosampler and DAD detector. Separation of flavonoids was performed on a Zorbax Eclipse XDB-C18 column (5 µm, 12.5 mm × 4.6 mm, Agilent), while arbutin was determined using a Nucleodur 100-5 C18 column (Macheray-Nagel, Düren, Germany), both combined with corresponding guard columns. HPLC standards and enzymes were purchased from Sigma-Aldrich (St. Louis, MO, USA). The purity of standards was 97% or higher. Methanol was HPLC grade. Other reagents and chemicals were of analytical grade.

### 3.2. Determination of Metal Content in Plant Material

Yttrium standard solution (10 µL, 1 g/L) was added to extract sample (1000 µL). After homogenization, 10 µL of the resulting solution was pipetted at the center of an unsiliconized quartz glass sample carrier, dried in a laboratory oven at temperature approximately 50 °C and put into the TXRF spectrometer. The measurement time was 1000 s. The concentration of the detected elements was estimated with respect to the known quantity of yttrium.

### 3.3. Preparation of the Extracts

Dried *V. myrtillus* leaves with stems (2 g) were milled, passed through a sieve of 850 µm mesh size and suspended in 20 mL of the solvent (water or 80% ethanol) in a 50 mL Erlenmeyer flask. The extraction was performed at 80 °C using an ultrasonication power of 720 W. After 30 min the suspensions were centrifuged for 30 min (3400 rpm) and the supernatant collected. The resulting solution was either evaporated at 30 °C on a rotavapor (hydroethanolic extract) or freeze-dried (aqueous extract).

### 3.4. Spectrophotometric Determination of Total Phenolic and Total Flavonoid Content

Total phenolic content (TP) and total flavonoid content (TF) were determined using Folin–Ciocalteau reagent [42] and aluminum chloride [43], respectively. TP and TF were calculated from calibration curves recorded for the standards as mg of standard equivalents in g of dry weight. Gallic acid and quercetin were used as standards for TP and TF, respectively (Table 2).

### 3.5. HPLC Analysis of Phenolic Constituents

Chromatographic standards (flavonoids and phenolic acids) and the extracts were dissolved in methanol in concentration of 0.2 mg/mL and 2 mg/mL, respectively. Solutions were filtered through a 0.45 µm PTFE syringe filter. Separation was performed at temperature of 40 °C and flow of 1.0 mL/min using following protocol: 0 min 20% B, 10 min 40% B, 35 min 50% B, where solvents A and B consisted of water, methanol and formic acid in proportions 93:5:2 (*v*:*v*:*v*) and 3:95:2 (*v*:*v*:*v*), respectively. Calibration curves of the standards were constructed using either the absorbance at 270 nm (hyperoside, kaempferol, protocatechuic acid and quercetin) or 320 nm (caffeic, chlorogenic and *p*-coumaric). Calibration curve parameters were determined according to [44] and reported in Table 3. The peak assignment and identification was based on comparison of retention times of peaks in sample chromatogram and UV spectra with those of the standards. For the analysis of flavonoid aglycones and bound phenolic acids, 1 mL of the extract solution in methanol was mixed with 400 μL of 6M HCl. The obtained mixtures were heated for 2 h in a boiling water bath and filtered to 5 mL volumetric flask. Methanol was added to the volume.

The presence of arbutin and hydroquinone was investigated using the pharmacopoeial monograph procedure for bearberry leaf [45], using standards of arbutin and hydroquinone in concentrations of 1.0 mg/mL and 0.25 mg/mL, respectively. Extracts were prepared in the concentration 10 mg/mL. Isocratic elution by methanol−water (10:90, *v*/*v*), flow rate of 1.2 mL/min and detection at 280 nm were used. For analysis 20 µL of the solutions were applied.

### 3.6. Total Antioxidant Activity

Total Antioxidant Activity (TAA) of extracts was determined according to [25]. Sample solution (0.1 mL) was combined with of reagent solution (1 mL) consisting of 0.6 M sulfuric acid, 28 mM sodium phosphate and 4 mM ammonium molybdate. The tubes tightly closed and incubated at 95 °C for 90 min. After cooling to room temperature, the absorbance at 695 nm was measured. Antioxidant activity was expressed as mg ascorbic acid equivalent (AAE) per g of dry weight based on the calibration curve of ascorbic acid.

### 3.7. Reducing Power *(*RP*)*

Reducing power (RP) was determined by the previously described spectrophotometric method [46]. 0.5 mL of phosphate buffer (0.2 M, pH 6.6) was mixed with potassium ferricyanide (0.5 mL, 1.0%) and extract solution (0.2 mL). The solution was incubated at 50 °C for 20 min and trichloroacetic acid (0.5 mL, 10%) was added. The obtained supernatant (0.5 mL) was mixed with distilled water (0.5 mL) and ferric chloride (0.1 mL, 0.1%). The absorbance of the final solution was read spectrophotometrically at 700 nm against a blank, and the reducing power was calculated based on the calibration curves of Trolox, expressed as mg Trolox equivalent (TE) per g of dry weight.

### 3.8. Ferric Reducing Antioxidant Power (FRAP)

According to Benzie and Strain [47], the FRAP reagent consisted of acetate buffer (25 mL, 300 mM), 2,4,6-tripyridyl-2-triazine solution (2.5 mL, 10 mM in 40 mM HCl) and ferric chloride solution (2.5 mL, 20 mM). To the FRAP solution (0.9 mL) the extract solution (0.1 mL) was added and left in the dark at 25 °C. After 30 min absorbance at 593 nm was read. FRAP was expressed as mg Trolox equivalent (TE) per g of dry weight based on Trolox calibration curve.

### 3.9. β-Carotene-Linoleic Acid Assay

The antioxidant activity of the substances was evaluated using the β-carotene-linoleic acid system according to modified literature procedures [48]. Tween 40 (200 mg) and β-carotene (1.0 mL, γ = 0.2 g/L) were mixed in chloroform. After removal of the solvent, linoleic acid (20 mg) and aerated distilled water (30 mL) were added. Aliquots (200 µL) of the emulsion were added to sample solutions in methanol (50 µL). After adding the emulsion to the sample solution, the reaction mixture was incubated at 50 °C for 120 min. During that period, the absorbance was measured at 450 nm at 15-min intervals. The percent of antioxidant activity (ANT) was calculated as RSA = (*R*_control_ − *R*_sample_)/*R*_control_ × 100, where *R*_control_ and *R*_sample_, are the average bleaching rates of the water control and antioxidant (test compound or BHA), respectively.

### 3.10. ABTS and DPPH Radical Scavenging Activity

ABTS (ABTS RSA) and DPPH (DPPH RSA) radical scavenging capacity were evaluated as described in [48,49], respectively. To appropriate concentration of the free radical solution, extract solution was added. After appropriate incubation at room temperature, absorbance was read at 734 nm or 545 nm for ABTS or DPPH free radical, respectively. RSA was calculated according to the equation RSA = (*A*_control_ − *A*_sample_)/*A*_control_ × 100 where *A*_control_ is the absorbance of the negative control (free solution without extract), and *A*_sample_ is the absorbance of the free radical solution containing extract. IC_50_ ABTS RSA and IC_50_ DPPH RSA were calculated as concentration of the extract which scavenges 50% of free radicals in the solution. Trolox and BHA were used as standard antioxidant for ABTS RSA and DPPH RSA, respectively.

### 3.11. Fe^2+^ Chelating Activity

For determining Fe^2+^ chelating activity (ChA) [48], methanolic extract solution (150 μL) was added to FeCl_2_ solution (0.25 mM, 50 µL) and incubated at room temperature for 5 min. Reaction was initiated by adding ferrozine solution (1.0 mM, 100 μL). After 10 min absorbance was measured at 545 nm. Negative control was reaction mixture with methanol (150 μL) instead of extract solution. ChA was calculated using *A*_control_ (absorbance of the negative control) and *A*_sample_ (absorbance of the solution with the extract). Chelating activity was calculated as IC_50_ ChA, the concentration that chelates 50% of Fe^2+^ ions. EDTA was used as chelating standard.

### 3.12. Determination of α-Glucosidase Inhibiting Activity

Inhibition of α-glucosidase was performed according to Tiwari et al. [50]. Extracts solutions (100 μL) in 10% DMSO were incubated at 37 °C with Type I α-glucosidase from *Saccharomyces cerevisiae* (50 μL, 1.0 U/mL in 0.1 M phosphate buffer, pH 6.8). After 10 min, *p*-nitrophenyl-α-d-glucopyranoside (PNG, 50 μL, 5 mM in the same buffer) was added. Absorbance at 405 nm was measured after 5 min against a blank solution where PNG was replaced with 50 μL of buffer. Control which represented 100% enzyme activity was prepared by replacing extract with 10% DMSO, while acarbose served as a positive control. Enzyme inhibition was calculated using the equation AG = (*A*_control_ − *A*_sample_)/*A*_control_ × 100 where *A*_control_ is the absorbance of the control mixture and *A*_sample_ represents absorbance of samples containing the extracts or acarbose. Applying convenient Concentration of the test sample necessary to inhibit 50% activity of the enzyme (IC_50_) was calculated using regression analysis.

### 3.13. Alpha-Amylase Inhibition Assay

The assay was performed according to [51]. To different concentrations of the extracts (25 μL), porcine α-amylase (25 μL, 0.5 mg/mL) in phosphate buffer (20 mM, pH 6.9) was added. After 10 min at 25 °C, 0.5% soluble starch solution in the same buffer (25 μL) was added. After additional 10 min at 25 °C, the reaction was stopped by adding 96 mM 3.5-dinitrosalicylic acid reagent (50 μL). The microplate was then placed in a boiling water bath for 5 min. Absorbance was measured at 540 nm. Percent of enzyme inhibition was calculated as described above.

### 3.14. Cell Culture and Treatment

Hep G2 cells from European Collection of Cell Cultures (ECACC, Salisbury, UK) were cultured in minimum essential media (MEM) supplemented with 10% (*v*/*v*) fetal bovine serum (FBS), 20 IU/mL penicillin and 20 µg/mL streptomycin. For the experiments, cells were seeded into six-well plates. Plates were changed to FBS-free medium 24 h before the assay. Negative control cells (C) were kept only in MEM medium which contained 5.56 mM glucose. Hyperglycemic conditions were induced by additional 30 mM glucose (positive control, D). For determining the influence of *V. myrtillus* extracts on GSH content in hyperglycemic conditions Hep G2 cells were treated with 30 mM glucose plus either 0.05 mg/mL (D-0.05) or 0.1 mg/mL (D-0.1). Reduced glutathione content was measured 24 h after glucose addition.

### 3.15. Reduced Glutathione Content (GSH)

Prior to GSH concentration measurement, Hep G2 cells were lysed using ultrasonication. To the supernatant, 5% trichloroacetic acid was added, and the mixture centrifuged again (3000 rpm, 10 min). To supernatant (100 µL) phosphate buffer (600 µL, 0.3 M, pH 7.4) and 2,2-dithiobisnitrobenzoic acid (50 µL) dissolved in the same buffer were added. The production of yellow colored 5-thio-2-nitrobenzoic acid was measured at 405 nm. Results were calculated using molar extinction coefficient of the product (14,150 cm^−1^ M^−1^) [52].

### 3.16. Statistical Analysis

The experiments were performed in triplicate. The results were expressed as mean ± SD. Statistical comparisons with the controls were made using one-way ANOVA followed by Dunnett’s post-hoc test, while the comparisons between samples was performed using *t*-test. *p* values < 0.05 were considered statistically significant. Statistical analyses were performed using the demo version of GraphPad Prism (GraphPad Software, La Jolla, CA, USA, www.graphpad.com).

## 4. Conclusions

*V. myrtillus* leaf extracts possess excellent antidiabetic properties, as demonstrated by the numerous assays performed in this study. Aqueous extract was able to restore glutathione concentration in Hep G2 cells subjected to glucose-induced oxidative stress. Hydroethanolic extract strongly inhibited α-glucosidase, equal to antidiabetic drug acarbose. Both solvents produced extracts with excellent antioxidant properties. Many of the observed biological properties may be attributed to hyperoside and chlorogenic acid, the main phenolic components of the investigated extracts. Even though the presented results partly explain the extent and popularity of *V. myrtillus* leaves use as a supportive therapy of diabetes, further studies are needed to assess the potential usefulness of the observed antioxidant, α-glucosidase inhibiting and GSH restoring effect in Hep G2 cells.

## Figures and Tables

**Table 1:** Phenolic constituents of *V. myrtillus* extracts before and after hydrolysis.

	Before Hydrolysis		After Hydrolysis	
	Aqueous Extract	Hydroethanolic Extract	Aqueous Extract	Hydroethanolic Extract
Arbutin (mg/g)	10.60	*n.d.*	*n.d.*	*n.d.*
Caffeic acid (mg/g)	*n.d.*	*n.d.*	*n.d.*	6.58
Chlorogenic acid (mg/g)	20.82	11.21	*n.d.*	*n.d.*
*p*-coumaric acid (mg/g)	0.32	0.46	*n.d.*	11.90
Protocatechuic acid (mg/g)	*n.d.*	*n.d.*	176.45	*n.d.*
Kaempferol (mg/g)	*n.d.*	*n.d.*	*n.d.*	3.45
Quercetin (mg/g)	*n.d.*	*n.d.*	24.75	85.64
Hyperoside (mg/g)	32.16	44.43	*n.d.*	*n.d.*

*n.d.*—not detected.

**Table 2:** Radical scavenging activity for ABTS (IC_50_ ABTS RSA) and DPPH (IC_50_ DPPH RSA) free radical, antioxidant activity in β-carotene-linoleate assay (ANT), chelating activity (ChA) and α-glucosidase activity (IC_50_ AG) of *V. myrtillus* extracts.

Extract	ANT	IC_50_ ABTS RSA	IC_50_ DPPH RSA	IC_50_ ChA	IC_50_ AG
	%	mg/mL	µg/mL	µg/mL	mg/mL
Aqueous extract	93.4 ± 2.3 ^A^	251.1 ± 11.1 ^A^	59.0 ± 1.7 ^A^	135.2 ± 6.3 ^A^	2.53 ± 0.21 ^A^
Hydroethanolic extract	48.6 ± 13.4 ^B^	252.2 ± 21.2 ^A^	17.8 ± 0.1 ^B^	391.5±10.4 ^B^	0.29 ± 0.02 ^B^
Standard	^a^ 95.1 ± 2.4 ^A^	^b^ 41.7 ± 2.2 ^B^	^a^ 5.9 ± 0.1 ^C^	^c^ 4.0 ± 0.3 ^C^	^d^ 0.50 ± 0.01 ^B^

Values are average of 3 replications ± SD. ^A–C^ Differences within column (samples connected by different capital letters are statistically different at *p* < 0.05). Standards: ^a^ BHA, ^b^ Trolox, ^c^ EDTA, ^d^ Acarbose.

**Table 3:** Retention time and calibration curve parameters for flavonoids and phenolic acids standards observed in chromatograms.

Standard	Calibration Curve Equation	*r*^2^	LOD (µg)	LOQ (µg)
Arbutin	y = 103.9x + 17.6	0.9999	0.005	0.016
Caffeic acid	y = 15197.0x + 199.3	0.9997	0.035	0.105
Chlorogenic acid	y = 2587.3x + 73.4	0.9996	0.036	0.110
*p*-coumaric acid	y = 5735.6x + 89.3	0.9999	0.005	0.015
Protocatechuic acid	y = 2415.8x + 17.6	0.9999	0.005	0.016
Kaempferol	y = 2802.8x + 21.6	0.9998	0.026	0.078
Quercetin	y = 2086.9x – 36.8	0.9998	0.027	0.083
Hyperoside	y = 1426.2x + 15.4	0.9999	0.013	0.040

LOD = level of detection; LOQ = level of quantification; y = Area under curve (mAU × s); x = amount of the standard (µg).
